# Supplementary material for: Deciphering the influence of Bacillus subtilis strain Ydj3 colonization on the vitamin C contents and rhizosphere microbiomes of sweet peppers
Source: PLoS One. 2022 Feb 28;17(2):e0264276. doi: 10.1371/journal.pone.0264276 (PMC8884494; doi:10.1371/journal.pone.0264276)
Supplement: Attachment — Submitted filename: renamed_c18b1.docx [file pone.0264276.s001.docx]

Line 89: add citation ” assessed for antagonistic activity against Colletotrichum gloeospoioides, Botrytis cinerea, and Phytophthora capsici by dual culture assay.”

Lines 220-223: Supplementary information on where the soil sample was collected, was it collected from the surface?

Fig. 1: Redraw the figure to facilitate understanding of groups and parts. Besides, the length of root hairs needs to be quantified.

Lines 342-348: Combined with the last part of the results, there may be a large number of other bacteria in the root system, so LB is not suitable for counting bacillus.

Lines 349-351: Due to the small sampling area of SCANNING electron microscopy, whether this result is repeated for many times. After all, a large number of bacteria were also detected in the last results (line 347: 5.85 ± 0.22 log10 CFU/g)

Fig. 2: Redraw to help understand groups. In addition, does the bacteria on the root surface caused pathological change?

Lines 364-385:

1. Please add more quality control information about 16s-hiseq.

2. The  form of FIG. 3 can only show the average gap between groups, but cannot show the inter-sample gap (standard deviation) within groups. Please add relevant information.

3. Whether similar results of ” antagonistic activity against Colletotrichum gloeospoioides, Botrytis cinerea, and Phytophthora capsici by dual culture assay (in the section materials and methods)” can be obtained through 16s-hiseq.
