# Supplementary material for: Deciphering the influence of Bacillus subtilis strain Ydj3 colonization on the vitamin C contents and rhizosphere microbiomes of sweet peppers
Source: PLoS One. 2022 Feb 28;17(2):e0264276. doi: 10.1371/journal.pone.0264276 (PMC8884494; doi:10.1371/journal.pone.0264276)
Supplement: Attachment — Submitted filename: PONE-D-21-26845 R1 Response to Reviewers.docx [file pone.0264276.s002.docx]

PONE-D-21-26845.

"Deciphering the influence of *Bacillus subtilis* strain Ydj3 colonization on the vitamin C contents and rhizosphere microbiomes of sweet peppers"

04-January-2022

Dear Dr. Ma and reviewers,

Thank you and the reviewer for the valuable comments to improve the manuscript!

We have addressed you and the reviewer’s comments in black text below, with our responses in blue. Line numbers noted below correspond to the revised manuscript file. We also include a revised manuscript text with track changes in the resubmission. Additionally, the manuscript was edited for proper English language, spelling and grammar by native English speaking editors at American Journal Experts (AJE), and the certificate was also included.

**Response to Editor:**
“**3. In your Methods section, please provide additional location information, including geographic coordinates of your field collection (for dragon Chinese juniper rhizosphere) if available.**”

We greatly appreciate you and the reviewer to point out what we have missed from the previous review, we had added the geographic information (23°91'93N, 120°51'33 E) of field collection in the description (line 83).

“**4. In your Methods section, please provide additional details regarding the dragon Chinese juniper used in your study, including ensuring you have described the source.**”

We didn’t use the dragon Chinese juniper in our study, we collected the soil from the rhizosphere of the dragon Chinese juniper.

“**5. Thank you for stating the following financial disclosure:
The research was financially supported by the Council of Agriculture, Taiwan [106AS-12.4.1-PI-P1, 110AS-5.4.2-PI-P2], the Ministry of Science and Technology, Taiwan [MOST 110-2321-B-005-006; 109-2321-B-005-022; 109-2313-B-005-032], and the “Innovation and Development Center of Sustainable Agriculture, NCHU” from the Featured Area Research Center Program within the framework of the Higher Education Sprout Project by the Ministry of Education in Taiwan. Please state what role the funders took in the study. If the funders had no role, please state: "The funders had no role in study design, data collection and analysis, decision to publish, or preparation of the manuscript." If this statement is not correct you must amend it as needed. Please include this amended Role of Funder statement in your cover letter; we will change the online submission form on your behalf.

Thank you for the instruction of the statement about the financial support. We have put the statement "The funders had no role in study design, data collection and analysis, decision to publish, or preparation of the manuscript." as suggested in the cover letter.

“6. Please include your full ethics statement in the ‘Methods’ section of your manuscript file. In your statement, please include the full name of the IRB or ethics committee who approved or waived your study, as well as whether or not you obtained informed written or verbal consent. If consent was waived for your study, please include this information in your statement as well. “

The statement “The field research and materials collected were under the agreement by the farm owner and granted permission by the Council of Agriculture, Taiwan.” was included in the “Method” section. (Lines 92-93)

**Response to Reviewer #1:**

“Line 89: add citation ” assessed for antagonistic activity against Colletotrichum gloeospoioides, Botrytis cinerea, and Phytophthora capsici by dual culture assay.”

The reference was cited in the main text (line 85) and included in the reference list [2].

“Lines 220-223: Supplementary information on where the soil sample was collected, was it collected from the surface?”

We collected the soil samples from the roots of sweet pepper. Some of the fibrous root will be cut and removed, and then the soil samples were collected.

The sentence was revised as “For the analysis of the soil microbial community composition for each treatment, untreated and *B. subtilis* Ydj3-treated rhizosphere soil samples were collected from the sweet pepper roots at the beginning of planting and at the end of fruit harvest and stored at −20 °C until further analysis.” (Lines 213-216)

“Fig. 1: Redraw the figure to facilitate understanding of groups and parts. Besides, the length of root hairs needs to be quantified.”

The figure was labeled as suggested, and the length of the root hairs was quantified and the descriptions were stated in the Material and Methods and Result sections as follows.

In the Material and Methods section, the sentences “For optical microscope observation, at least ten root hairs from different zones for each treatment were randomly chosen and their lengths were measured.” were added (lines 168 to 170).

In the Results section, the sentences “The lengths of root hairs in each treatment was measured using the software (cellSens Standard 1.9) installed in the Olympus Fluorescence BX60 microscope, and results showed that the length of root hair of B. subtilis Ydj3 treatment had was longer than in the Ctrl treatment, with an average length in the apical meristem of 279.4 ± 177.3 µm compared with the Ctrl treatment , with an average length of 61.8 ± 8.8 µm; an average length of 375.9 ± 143.0 µm in the elongation zone, significantly longer than the Ctrl treatment, with an average length of 120.6 ± 63.4 µm; and an average length of 491.4 ± 21.9 µm in the maturation zone, significantly longer than the Ctrl treatment, with an average length of 172.4 ± 17.6 µm.” were added. (lines 318 - 326)

“Lines 342-348: Combined with the last part of the results, there may be a large number of other bacteria in the root system, so LB is not suitable for counting bacillus.”

We agree with the reviewer that there are a large number of bacteria in the root system. The Bacillus and other bacteria were differentiated by colony morphology on the LB agar plates as shown in the following figure and the Bacillus-like colonies were randomly picked for the identification by 16S RNA sequencing. We found that the colonies obtained from the treatment with *B. subtilis* Ydj3 were all *Bacillus* species. The material and method and the statement in the result section was revised in the text (lines 180-182 and 336-342, respectively).

(A)
 (B)

Fig. The colonies obtained from root samples of (A) the control; and (B) the *B. subtilis* Ydj3 treatment.

“Lines 349-351: Due to the small sampling area of SCANNING electron microscopy, whether this result is repeated for many times. After all, a large number of bacteria were also detected in the last results (line 347: 5.85 ± 0.22 log10 CFU/g)”

As stated in the above reply, the Bacillus and other bacteria were differentiated by colony morphology on the LB agar plates, and the Bacillus-like colonies were randomly picked for the identification of *Bacillus* species by 16S RNA sequencing.

“Fig. 2: Redraw to help understand groups. In addition, does the bacteria on the root surface caused pathological change?”

We have labeled respective treatment in the figure as suggested. We did not observe pathological effect on the root caused by *B. subtilis* Ydj3, a plant growth rhizobacterium (PGPR). The change in root architecture- enhancing root hair formation may be partly due to the production of indole-3-acetic acid (17 mg/L) by *B. subtilis* Ydj3 (our unpublished data and discussed in lines 423-425). Similarly, Richardson et al. (2009) indicated that PGPR could change the root architecture via production of plant hormone.

“Lines 364-385:

1. Please add more quality control information about 16s-hiseq.”

The cutoff value of the bacterial communities in the rhizosphere soil of sweet pepper plants is 97%, and the Effective Tags Q20 (%) and Q30 (%) values of each treatment showed below were all above 85%.

| treatment | Q20 (%) | Q30 (%) |
| --- | --- | --- |
| Ctrl 0 | 91.36 | 87.10 |
| Ctrl 1 | 90.24 | 85.40 |
| Ydj3 | 91.31 | 86.90 |

Additionally, the description of the quality control was also stated in the main text as follows: “The Q20 values (%) of Ctrl 0, Ctrl 1 and Ydj3 were 91.36%, 90.24% and 91.31%, respectively, and the Q30 values (%) of Ctrl 0, Ctrl 1 and Ydj3 were 87.10%, 85.4% and 86.90%, respectively.” (Lines 362 - 365).

“2. The form of FIG. 3 can only show the average gap between groups, but cannot show the inter-sample gap (standard deviation) within groups. Please add relevant information.”

The main focus of the study was to compare the compositions of bacterial community between the control and the *B. subtilis* Ydj3 treatment, thus the inter-sample gap (standard deviation) within groups were not provided.

“3. Whether similar results of ” antagonistic activity against *Colletotrichum gloeospoioides*, *Botrytis cinerea*, and *Phytophthora capsici* by dual culture assay (in the section materials and methods)” can be obtained through 16s-hiseq.”

Thanks for the suggestion. The effect on the compositions of fungal community by the *B. subtilis* Ydj3 treatment will be investigated in the future.

**Response to Reviewer #2:**

“Line 25 – I don’t think the term “priming” is adequate here. Please rephrase.”

The term “ priming” was deleted as suggested. The statement was revised as “The promotion of seed germination was observed for sweet pepper seeds treated with the Ydj3 strain, indicating that Ydj3 promoted seed germination and daily germination speed (131.5 ± 10.8 seeds/day) compared with the control (73.8 ± 2.5 seeds/day).” (Lines 22 - 24)

“Line 108 – Please include the abbreviation of germination value (GV).”

The abbreviation of germination value (GV) was added. (Line 103)

“Line 118 and 120 – Seedlings are young plants, and so after 40 days of treatment the correct designation should be “plants”.

Line 139 – As described above, correct? ”

They were revised as suggested. (Lines 112 and 113)

“Line 165 – Zhang et al. (2014) is reference [27].”

The reference was cited in the main text (Line 156) and the reference list no [32] was revised accordingly.

“Lines 225-226 – Is this a repetition of the above? (lines 221 - 222)?”

The repeated sentence was deleted.

“Line 278 – Did you mean germination value (instead of daily germination speed)?”

Our results indicated that the *B. subtilis* Ydj3 treatment exhibited higher daily germination speed (DGV) than the control, so that the mean germination value (MGT) was shortened. (Lines 260-266)

“Line 283-284 (Table 1) – Please indicate units for mean germination time, germination value and daily germination speed. Correct the abbreviation of daily germination speed (DGV, not DGP). Are columns “Germination value” and “Daily germination speed” swapped?”

It was revised as suggested and the unit of each term was added in Table 1. (Table 1)

The unit of mean germination time (MGT) is day, and there is no unit of germination value (GV), GV is an index and calculated by the formula cited in reference no [6]. The unit of daily germination speed (DGV) is seeds/day, and the abbreviation was also revised.

“Line 304-305 – These are the total fruit yield values, not by group, right? If yes, remove /group.”

It was revised as suggested and the term “/group” was deleted. (Lines 297 - 298)

“Line 320 (Table 2) – Total yield: Same as in the previous comment. Large fruits, medium fruits, small fruits: did you mean number of fruits/group (average of three replicates)? Lee et al. is reference [12].”

It was revised as suggested. In Table 2, the unit of total yield was kg, the unit of large fruits, medium fruit and small fruits was revised as “numbers of fruits”. The reference was added in Table 2 and the reference list no [16] (Line 305).

“Line 331 – showed that the distribution”

The description was deleted. (Line 313)

“Line 338 (Fig 1) – Are the captions of (A,D) and (C,F) swapped?”

The figure and figure legend were revised as suggested. (A, D) are apical meristem, (C, F) are maturation zone. (Fig 1.) (Lines 330-332)

“Line 368 – “A total of 16 distinct bacterial phyla”: Is this correct number of distinct phyla?”

It was revised as “ A total of 17 distinct bacterial phyla.” (Line 365)

“Line 404 – 25% increase in seed germination: how was this value obtained and what parameter does it refer to?”

The values was obtained as follows: GP (%) of Yjd3 – GP (%) of Ctrl =91.7% - 66.7% = 25%. (Line 398)

“Line 405 -… compared with the average value of control treatments”

The sentence was revised as suggested. (Lines 399-400)

“Line 388 (Fig 3 caption) – (A) Relative abundances of most abundant phyla?”

The Fig 3 caption was revised as suggested. (Line 382)

**Response to Reviewer #3:**

“Page 5, line 55: Perhaps the authors can add more up-to-date citations”

The reference was updated to the statistical data in 2020 in the main text (Line 52) and the reference list no [8] (Lines 498-499).

“Material and Methods
Microscopy observation and root colonization, Page 11 and 12
Please add number of replicates, number of plants per replicate, number of analyzed images.”

For the assay, each treatment contained 5 plants and at least 3 images were taken at different magnification for each sample.

The statement was revised as the follows accordingly.

“For each treatment, five plant roots were observed using optical microscopy and scanning electron microscopy. “ (Lines 162-163)

“For optical microscope observation, at least ten root hairs from different zones for each treatment were randomly chosen and their lengths were measured.” (Lines 168 – 170)

“Each sample was observed at 2000X, 3000X and 4000X magnification and at least 3 images were taken for analysis.” (Lines 175 - 176)

“Results
Page 21, line 338: In the description of figure 1, the letters are wrong, (A, D) is the meristematic zone and (C, F) is the zone of differentiation.
Commonly, the hairy area is called the differentiation zone, perhaps a more in-depth study of the state of differentiation of cells in these areas that present root hairs with treatment with the bacteria should be carried out. Perhaps the meristematic and elongation zones are more apical.”
The legend of Fig 1 was corrected as suggested. (A,D), apical meristem; (B, E) elongation zone; (C,F) maturation zone. (Lines 330-332)

“Page 22, line 353: A more detailed description of figure 2 would be desirable, indicating that it is observed in the images.
In image A, cells are observed, in B, the apex of a root (it would be desirable to indicate the bacteria) and in C, the root hairs (it would be desirable to indicate in the image which are the root hairs and which it's bacteria).”
It was revised as suggested (Lines 347-350). The revised image was attached with the revised file.

“Discussion
If possible, explain in more detail the non-variation of the dry weight of the root between treatments (table 1), although the architecture of the root varies, increasing the root hairs in those roots that are treated with the bacteria.”

The discussion was added as stated “understanding and quantifying the impact of PGPR on roots and the whole plant remain challenging. Several studies indicated that PGPR modified the root structure including primary root growth, lateral root density, root dry weight, however, the root dry weight was not enhanced in our results. This might be because the plants were young and the variation remained non-significant.” (Lines 406 - 410)

Thank you again for giving us the opportunity to address the question!

Sincerely,

Tzu-Pi

***********************************************************

Tzu-Pi Huang, Ph. D.

Professor, Department of Plant Pathology

Director, Pesticide Residue Analysis Center

National Chung Hsing University

145, Xing-Da Road, Taichung 40227, Taiwan

TEL: 886-4-22840780 ext. 379; 886-4-22859750

FAX: 886-4-22859750

E-mail: [tphuang@nchu.edu.tw](mailto:tphuang@nchu.edu.tw)

***********************************************************
