# Supplementary material for: Deciphering the influence of Bacillus subtilis strain Ydj3 colonization on the vitamin C contents and rhizosphere microbiomes of sweet peppers
Source: PLoS One. 2022 Feb 28;17(2):e0264276. doi: 10.1371/journal.pone.0264276 (PMC8884494; doi:10.1371/journal.pone.0264276)
Supplement: Attachment — Submitted filename: PONE-D-21-26845 R2 Response to Reviewers.docx [file pone.0264276.s003.docx]

PONE-D-21-26845.

"Deciphering the influence of *Bacillus subtilis* strain Ydj3 colonization on the vitamin C contents and rhizosphere microbiomes of sweet peppers"

07-Febuary-2022

Dear Dr. Ma and reviewers,

Thank you and the reviewer for the valuable comments to improve the manuscript!

We have addressed the reviewer’s comments in black text below, with our responses in blue. Line numbers noted below correspond to the revised manuscript file. Additionally, a revised manuscript text with track changes and an unmarked version were included in the resubmission.

“Journal Requirements:
Please review your reference list to ensure that it is complete and correct. If you have cited papers that have been retracted, please include the rationale for doing so in the manuscript text, or remove these references and replace them with relevant current references. Any changes to the reference list should be mentioned in the rebuttal letter that accompanies your revised manuscript. If you need to cite a retracted article, indicate the article’s retracted status in the References list and also include a citation and full reference for the retraction notice.”

We also have reviewed and checked again the reference list and confirmed all the references cited are correct and have not been retracted. The reference cited in the statement “…fertilizer use [11], macronutrient and…” was changed to “…fertilizer use [10], macronutrient and…” (line 54).

**Response to Reviewer #2:**
“**1. Section "Fruit quality and vitamin C content" (Lines 130-132): Field experiment and growth conditions were described in the previous section. Therefore, "as described above" should be added at the end of the first sentence of this section, or it seems to refer to another experiment.**”

We greatly appreciate you and the reviewer to point out what we have missed from the previous review, we had added the description “as described above” at the end of the sentence (line 132-133).

“**2. Table 1. The position of the columns DGV and GV has been changed, but the contents are as in the previous version and are not in agreement with the text (lines 263-264). Please correct.**”

We had revised the data in the text respectively to Table 1 (line 263-264).

“**3. Table 2. The text in the first column should be unified. I suggest using "Number of large fruits" or "Mean number of large fruits" instead of "Large fruits (mean numbers of fruits)", and "Weight of large fruits" instead of "Large fruit weight". The same applies to medium fruits and small fruits. This will make Table 2 easier to read. **”

It was revised as suggested. (Lines 298-299 and line 307 Table 2)

“**4. Lines 314-316 – Suggestion: “Roots treated with *B. subtilis* Ydj3 presented denser root hairs at apical meristem, elongation zone, and maturation zone than those in the control (Fig 1). **”

It was revised as suggested (lines 316-317).

**Response to Reviewer #3:**

“**In any case, I do not agree with the concept that there are root hairs in all root zones (apical, elongation and differentiation/maturation), according to what is incorporated in the new version of the work (lines 318-326).
Root hairs are found in the zone of differentiation/maturation only. Perhaps the authors will find bibliography that supports their findings. **”

Thank you again for giving us the opportunity to address the question. Root hairs are cylindrical extensions of root epidermal cells that are important for acquisition of nutrients, microbe interactions, and plant anchorage. As descripted by Grierson et al. in the Arabidopsis Book (2014 (12); doi: [10.1199/tab.0172](https://dx.doi.org/10.1199%2Ftab.0172)), root hair development is regulated by auxin and ethylene, especially by auxin homeostasis. Increased auxin or ethylene signaling resulted in **moving the initiation site of root hair growth to a more apical position** and **increasing the amount of elongation during root hair tip growth**. Decreased auxin or ethylene signaling showed the opposite effects. Additionally, as stated in the discussion section of our manuscript, Vacheron et al. indicated that many PGPR affect root system architecture via modulation of host gormonal balance, including the plant auxin pathway (lines 418-420). In our study, we observed the formation of root hairs initiated at apical zone, however, the mechanisms underlying the interaction between the *B. subtilis* Ydj3 treatment and the root hairs growth of sweet peppers remain to be investigated in the future.

Thank you again for giving us the opportunity to address the questions!

Sincerely,

Tzu-Pi

***********************************************************

Tzu-Pi Huang, Ph. D.

Professor, Department of Plant Pathology

Director, Pesticide Residue Analysis Center

National Chung Hsing University

145, Xing-Da Road, Taichung 40227, Taiwan

TEL: 886-4-22840780 ext. 379; 886-4-22859750

FAX: 886-4-22859750

E-mail: [tphuang@nchu.edu.tw](mailto:tphuang@nchu.edu.tw)

***********************************************************
